# Supplementary material for: Spontaneous Neurotransmitter Release Shapes Dendritic Arbors via Long-Range Activation of NMDA Receptors
Source: Cell Rep. 2015 Feb 13;10(6):873–82. doi: 10.1016/j.celrep.2015.01.032 (PMC4542315; doi:10.1016/j.celrep.2015.01.032)
Supplement: Document S1. Figures S1–S3 [file mmc1.pdf]

Cell Reports

Supplemental Information

Spontaneous Neurotransmitter Release Shapes Dendritic Arbors via Long-Range Activation of NMDA Receptors

Laura C. Andreae and Juan Burrone

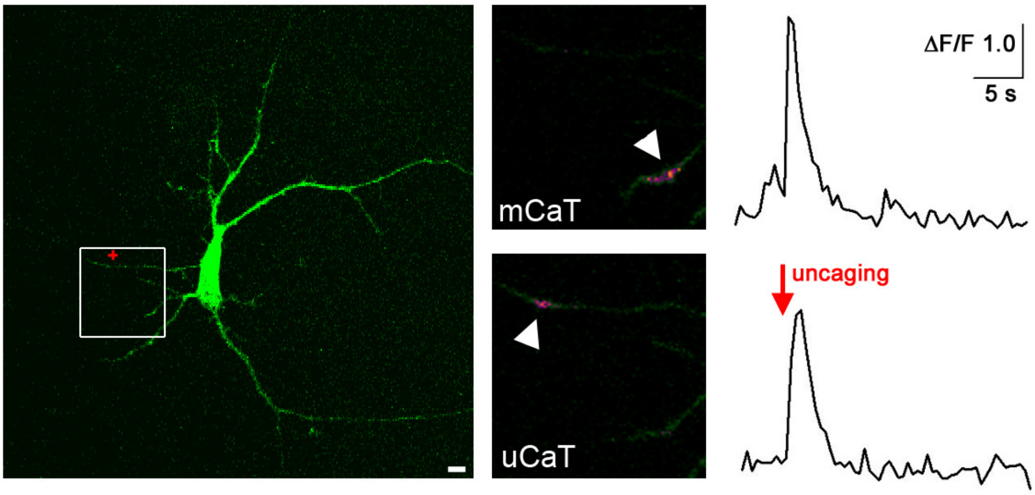

Figure S1, related to Figure 1. Spontaneous and uncaging-induced calcium events in immature dendrites of the same 4 DIV neuron. Left: GCaMP3-expressing neuron, uncaging site indicated (+). Middle panels show high-power views of boxed area, arrowheads indicate response sites, top: example spontaneous mCaT, bottom: uncaging-induced calcium transient (uCaT), corresponding ΔF/F traces to right. Scale bar 5 μm.

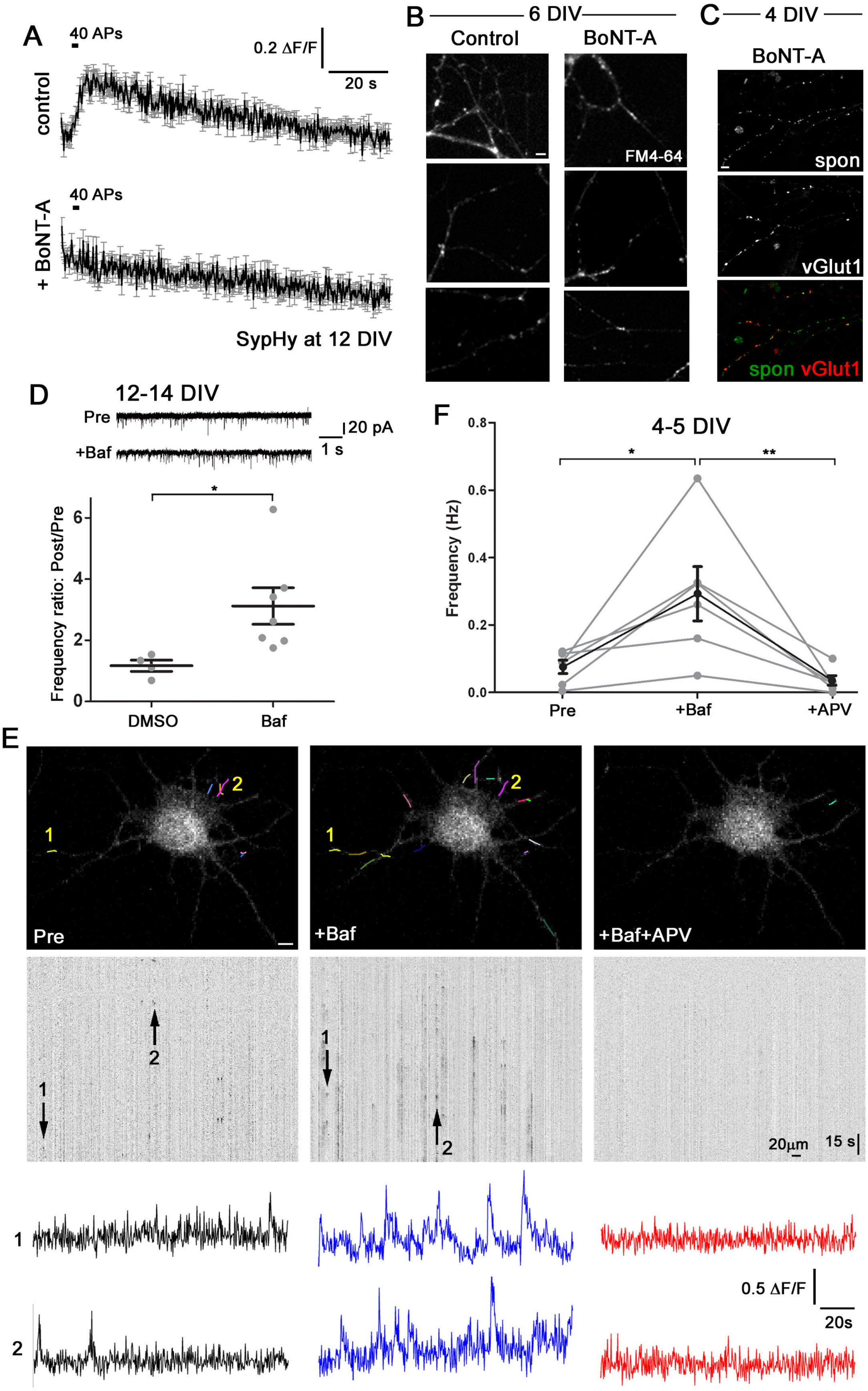
A
40 APs
0.2 ΔF/F
20 s
control
+ BoNT-A
40 APs
SypHy at 12 DIV
B
6 DIV
Control
BoNT-A
FM4-64
C
4 DIV
BoNT-A
spon
vGlut1
spon vGlut1
D
12-14 DIV
Pre
+Baf
20 pA
1 s
Frequency ratio: Post/Pre
DMSO
Baf
F
4-5 DIV
Frequency (Hz)
Pre
+Baf
+APV
E
Pre
+Baf
+Baf+APV
20μm
15 s
0.5 ΔF/F
20s

Figure S2, related to Figure 3. (**A**) In mature (12 DIV) neurons expressing the vesicle cycling reporter SypHy, incubation in 100 ng/ml Botulinum toxin A (BoNT-A) for 48 hrs leads to abolition of the SypHy response to field stimulation of 40 APs at 20Hz, confirming expected activity of the toxin. (**B**) However, similar incubation in BoNT-A has no obvious effect on spontaneous vesicle cycling at 6 DIV, labelled using the styryl dye FM-4-64: 3 representative images for each condition are shown, all taken using identical settings. Similar data was obtained at 3 DIV (not shown). (**C**) Example neuron at 4 DIV expressing biosyn after incubation in BoNT shows robust labelling of spontaneously cycling vesicles (spon); neuron shown is excitatory as indicated by colabelling with vGlut1, so lack of effect from BoNT-A is not due to inhibitory cell identity. (**D**) Electrophysiological recordings in mature neurons demonstrate a significant increase in mEPSC frequency following acute bath application of 1.6 mM Bafilomycin. (**E**) Example neuron at 5 DIV. Top panels: all spontaneous mCaTs shown in random colours, middle panels: corresponding kymograph with 2 examples indicated by arrows, lower panels: $\Delta F/F$ traces for examples): From left to right: baseline spontaneous activity, following bafilomycin application, after subsequent APV (same example ROIs throughout). (**F**) Quantification confirms a significant increase in mCaT frequency following Bafilomycin which is abolished by addition of APV at 4-5 DIV. Error bars represent sem. Scale bars 5 µm.

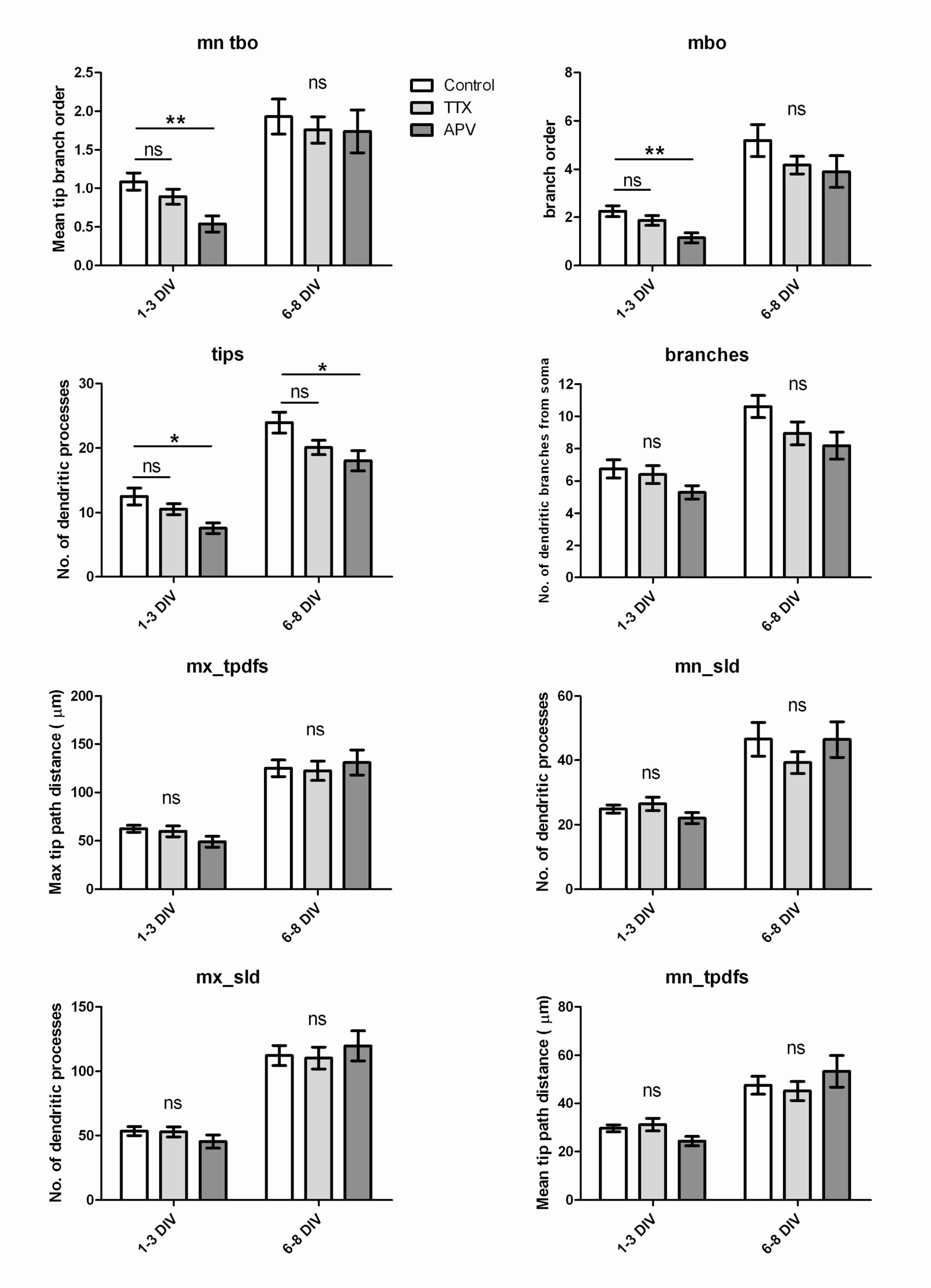

Figure S3, related to Figure 5. Quantification of other aspects of dendritic arbor formation following 48 hrs of TTX or APV treatment at different developmental timepoints. Error

bars represent sem. mn tbo: mean tip branch order, mbo: maximum branch order of any tip (eg. 0=soma branch, 1=1st bifurcation, etc.), tips: number of dendritic processes, branches: number of soma branches, mx_tpdfs: maximum distance from any tip back to soma, along dendrites, mn_sld: mean distance from tip to soma, straight line, mx_sld: maximum distance from tip to soma, straight line, mn_tpdfs: mean distance from tip back to soma, along dendrites.
