# Supplementary material for: Spontaneous Neurotransmitter Release Shapes Dendritic Arbors via Long-Range Activation of NMDA Receptors
Source: Cell Rep. 2015 Feb 13;10(6):873–82. doi: 10.1016/j.celrep.2015.01.032 (PMC4542315; doi:10.1016/j.celrep.2015.01.032)
Supplement: Document S2. Article plus Supplemental Information [file mmc2.pdf]

Cell Reports

Report

Spontaneous Neurotransmitter Release Shapes Dendritic Arbors via Long-Range Activation of NMDA Receptors

Graphical Abstract

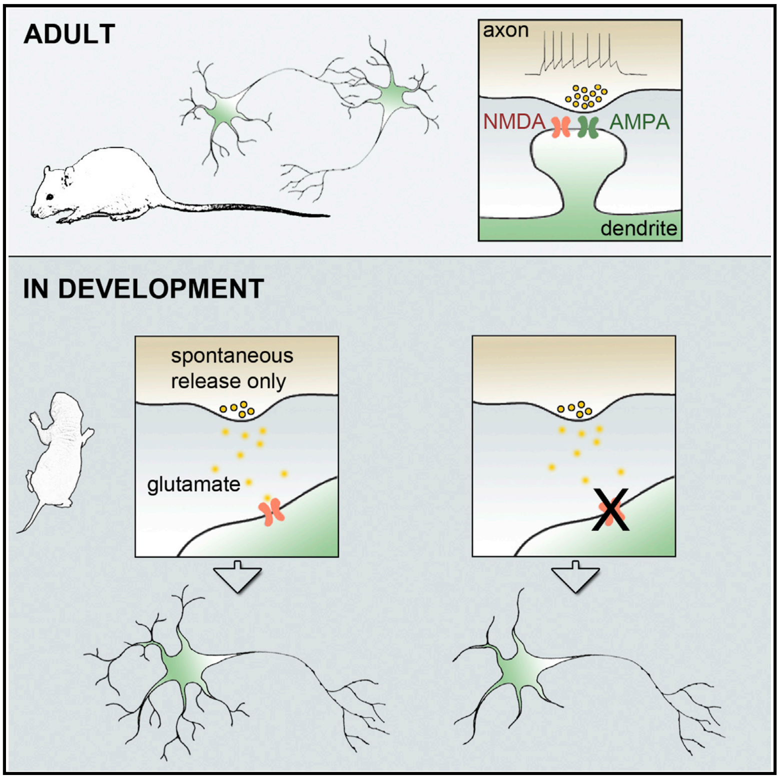

Highlights

- New role for spontaneous neurotransmitter release in dendritic arbor formation
- Vesicular glutamate can activate distant "long-range" dendritic NMDA receptors
- Presynaptic and postsynaptic machinery operate before synaptic contact
- Spontaneous glutamate release may provide local dendritic guidance or branching cue

Authors

Laura C. Andreae, Juan Burrone

Correspondence

laura.andreae@kcl.ac.uk (L.C.A.),
juan.burrone@kcl.ac.uk (J.B.)

In Brief

Spontaneous neurotransmitter release is a core element of synaptic communication in mature neurons, but little is known of its function during neuronal development. Andreae and Burrone now show that spontaneous glutamate release exerts a specific role prior to synapse formation to control dendritic arbor formation, signaling via "long-range" activation of NMDA receptors.

CrossMark

Andreae & Burrone, 2015, Cell Reports *10*, 873–882
February 17, 2015 ©2015 The Authors
http://dx.doi.org/10.1016/j.celrep.2015.01.032

CellPress

Cell Reports
Report

OPEN ACCESS
CellPress

Spontaneous Neurotransmitter Release Shapes Dendritic Arbors via Long-Range Activation of NMDA Receptors

Laura C. Andreae[1,*] and Juan Burrone[1,*]
[1]MRC Centre for Developmental Neurobiology, King's College London, London SE1 1UL, UK
*Correspondence: laura.andreae@kcl.ac.uk (L.C.A.), juan.burrone@kcl.ac.uk (J.B.)
http://dx.doi.org/10.1016/j.celrep.2015.01.032
This is an open access article under the CC BY license (http://creativecommons.org/licenses/by/3.0/).

SUMMARY

Spontaneous neurotransmitter release is a core element of synaptic communication in mature neurons, but despite exceptionally high levels of spontaneous vesicle cycling occurring in developing axons, little is known of its function during this period. We now show that high-level, spontaneous axonal release of the neurotransmitter glutamate can signal at long range to NMDA receptors on developing dendrites, prior to synapse formation and, indeed, axodendritic contact. Blockade of NMDA signaling during this early period of spontaneous vesicle cycling leads to a reduction in dendritic arbor complexity, indicating an important role for early spontaneous release in dendritic arbor growth.

INTRODUCTION

The spontaneous release of neurotransmitter is thought to have important functions in modulating activity-dependent transmission and synaptic homeostasis (Frank et al., 2006; Lee et al., 2010; Sutton et al., 2006). It is increasingly clear that vesicles undergoing spontaneous release represent a molecularly (Ramirez et al., 2012) and functionally (Chung et al., 2010; Fredj and Burrone, 2009; Sara et al., 2005) distinct population. Moreover, we previously demonstrated that immature (4–5 days in vitro [DIV]) cultured hippocampal neurons display very high levels of exclusively spontaneous vesicle cycling that derives from a functionally separate vesicle pool throughout development (Andreae et al., 2012), suggestive of an independent developmental role.

It is well known that neuronal activity in the form of classical action potential firing is important for the development of neuronal circuits, particularly in circuit refinement and maintenance (Bleckert and Wong, 2011). Although synapses per se are still generated in the absence of synaptic transmission (Verhage et al., 2000), it has long been hypothesized that activity might in some way regulate synaptogenesis. This is supported by observations on the behavior of highly motile dendritic filopodia during development, which can be precursors to mature synapses (Ziv and Smith, 1996). In addition, interfering with neurotransmission affects dendritic filopodial number, shape, and motility (Portera-Cailliau et al., 2003; Wong and Wong, 2001). Dendritic filopodia also mediate dendritic growth and branching, potentially via filopodial stabilization and synapse formation (Cline and Haas, 2008; Dailey and Smith, 1996; Niell et al., 2004; Vaughn, 1989), and indeed, modulation of neuronal activity in *Xenopus* tectal neurons impacts on dendritic arbor formation (Rajan and Cline, 1998; Rajan et al., 1999; Sin et al., 2002). Historically, studies have mostly focused on evoked (activity-dependent) neurotransmission, although early evidence indicated that spontaneous (activity-independent) transmitter release was involved in the maintenance of dendritic spines (McKinney et al., 1999) and could prevent the growth-promoting effects of brain-derived neurotrophic factor (BDNF) on basal dendrites of cortical neurons (McAllister et al., 1996). Recently, work at the *Drosophila* larval neuromuscular junction has clearly demonstrated that spontaneous transmission alone can play a critical role in presynaptic bouton size and maturation (Choi et al., 2014). However, it has been difficult to separate out events that might occur prior to axodendritic contact from those that are dependent on contact.

In this study, we have been able to take advantage of our in vitro system to dissect out the specific contribution of activity-independent, spontaneous neurotransmission prior to axodendritic contact in vertebrate neurons of the central nervous system. We show that in this system the majority of synaptic vesicles cycling spontaneously in developmentally immature axons express the glutamate transporter vGlut1, providing strong evidence that they release the excitatory neurotransmitter glutamate. Using the genetically encoded calcium indicator, GCaMP3, we find that this spontaneously released glutamate can be detected by "long-range" NMDA receptors localized on distant dendrites. We have previously shown that at this early stage (4–5 DIV) neurons do not undergo activity-dependent vesicle cycling, which initiates later in development. We therefore antagonized NMDA-dependent signaling in different developmental time windows and found that blockade during the early period of exclusive spontaneous vesicle cycling significantly reduced dendritic arbor complexity. At the same stage, preventing evoked release with tetrodotoxin (TTX) had no effect. Hence, our results indicate a new important role for spontaneous neurotransmitter release early in development on dendritic arbor formation and branching.

OPEN
ACCESS
CellPress

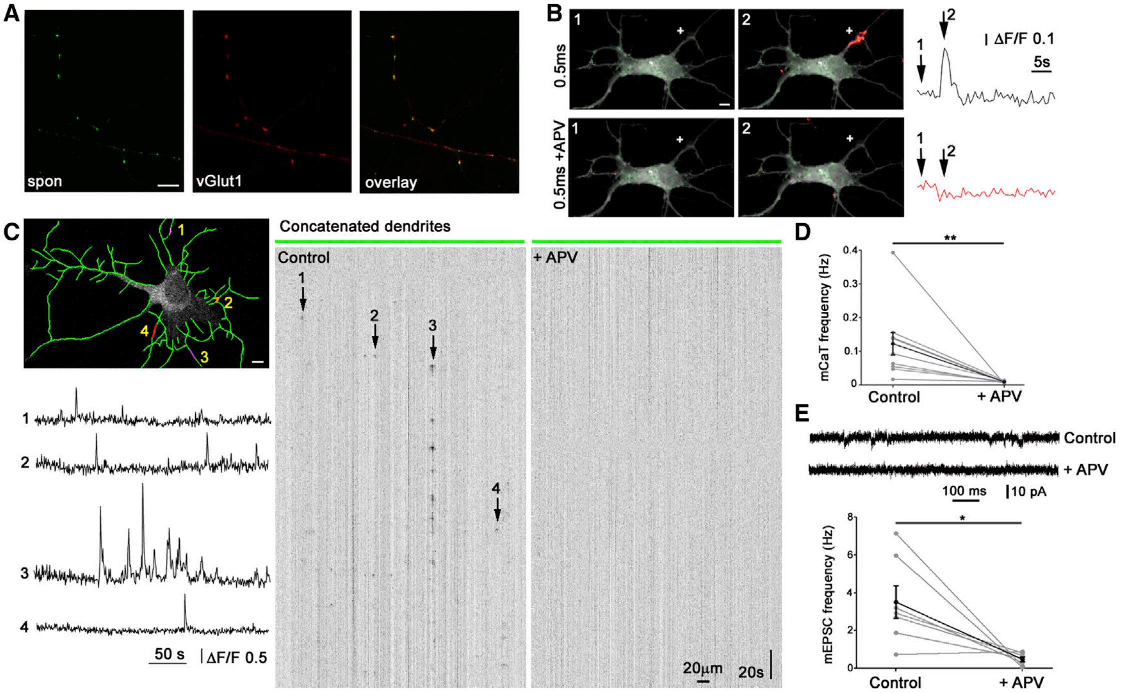

Figure 1. Immature Neurons Show Spontaneous NMDA-Dependent Events

(A) Biosyn labeling of spontaneously cycling VAMP2^{+} vesicles (spon) colocalizes with vGlut1 staining at 4 DIV.

(B) Focal glutamate uncaging (+ on image) induces a robust localized calcium transient in a 4 DIV dendrite expressing GCaMP3 in 0 Mg^{2+} (top, "Blue Orange icb" lookup table, ImageJ). This transient is abolished by application of APV (50 μM, bottom).

(C) Example of spontaneous mCaTs in a single neuron imaged over 300 s. Tracings obtained in NeuronJ are overlaid in green with example responses superimposed (numbered 1–4). Tracings were concatenated to obtain kymographs showing normalized fluorescence for all dendrites as a function of time (see Experimental Procedures). Example mCaTs are indicated by arrows, with corresponding ΔF/F traces shown to the left; all are abolished by APV.

(D) Quantification shows that spontaneous mCaTs at 4–5 DIV are reduced by APV (**$p < 0.01$, n = 10 cells).

(E) Electrophysiological recordings at 4–5 DIV demonstrate spontaneous NMDA mEPSCs, which are abolished by APV (*$p < 0.05$, n = 7).

Error bars represent SEM. Scale bars are 5 μm. See also Figure S1.

RESULTS

Young Neurons Release Glutamate and Corresponding Dendrites Show Spontaneous NMDA-Dependent Events

Synaptogenesis has been well described to occur in dissociated hippocampal neurons between 7 and 12 DIV (Basarsky et al., 1994; Renger et al., 2001), and mobile presynaptic transport vesicles in cortical neurons are known to already contain the vesicular glutamate transporter vGlut1 (Sabo et al., 2006). To identify the predominant transmitter type released spontaneously by immature axons prior to synapse formation in our system, we labeled spontaneously cycling vesicles in 4–5 DIV dissociated hippocampal neurons with biosyn (Andreae et al., 2012), followed by post hoc immunolabeling against vGlut1. This indicated that the majority of vesicles (mean, 73% [130/178 boutons] ± 8.7%, n = 6 cells) was likely to release the excitatory neurotransmitter glutamate (Figure 1A). VGlut2 labeled only a subset of vGlut1^{+} puncta (data not shown). During synapse maturation, postsynaptic NMDA glutamate receptors often precede α-amino-3-hydroxy-5-methyl-4-isoxazolepropionic acid (AMPA) receptors (Lin and Constantine-Paton, 1998; Wu et al., 1996) and indeed undergo membrane cycling prior to synapse formation (Renger et al., 2001; Washbourne et al., 2004). Therefore, to identify a potential postsynaptic receptor, we used focal glutamate uncaging (in the presence of TTX and absence of Mg^{2+}) to reveal possible NMDA-mediated calcium transients. The dendrites of neurons expressing the genetically encoded calcium sensor GCaMP3 showed robust, localized Ca^{2+} transients in response to uncaging pulses (0.5–1 ms long) previously shown to evoke postsynaptic currents of quantal amplitude (Kay et al., 2011). These were abolished by the NMDA antagonist D-(-)-2-Amino-5-phosphonopentanoic acid (APV) (Figure 1B), indicating that highly immature dendrites have the potential to respond to glutamate in a spatially discrete way that is NMDA dependent.

To determine whether we could detect the dendritic responses to early spontaneous glutamate release, we performed time-lapse imaging of GCaMP3-positive neurons under the same conditions and were able to detect miniature Ca^{2+} transients (mCaTs), which localized to discrete dendritic regions and were significantly reduced by APV ($p < 0.01$; Figures 1C, 1D, and S1). Residual localized dendritic calcium transients that were NMDA independent were usually seen after APV treatment, which have been previously described (Lohmann and Bonhoeffer, 2008). The kinetics of these spontaneous mCaTs were not significantly different from those due to uncaging (mean rise time: spontaneous 2.06 s, uncaging 2.28 s,

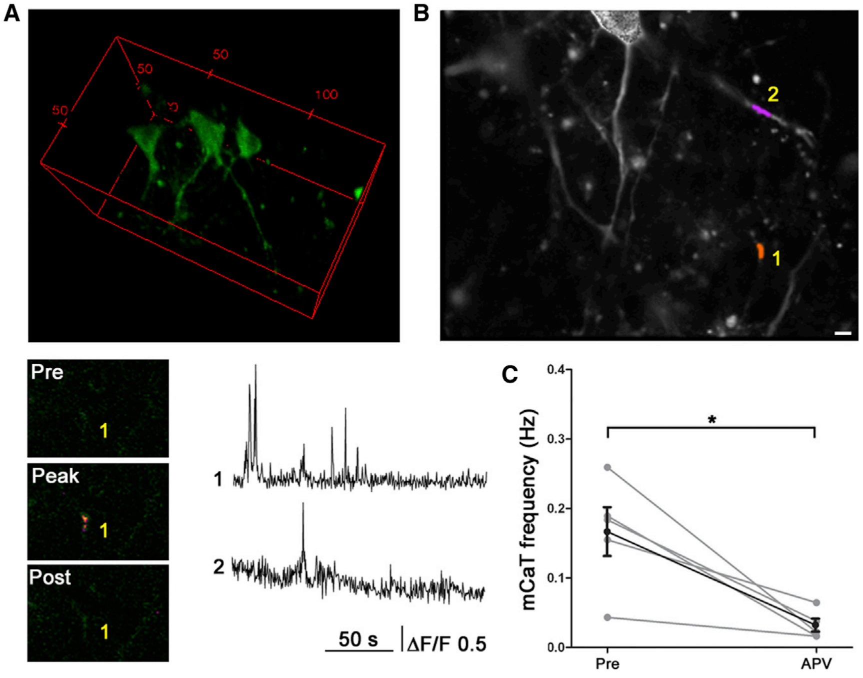

Figure 2. Spontaneous NMDA-Dependent mCaTs in Immature Hippocampal Slices

(A) 3D projection of example region of P4 hippocampal slice showing neurons infected with AAV-GCaMP5, axes in microns.

(B) Two example mCaT responses: locations indicated (orange and pink) with time-lapse images of site 1 below, showing the frame before peak response, peak, and 740 ms after peak. Corresponding ΔF/F traces (1 and 2) to the right.

(C) Quantification shows that spontaneous mCaT frequency at P3–P4 is reduced by APV (* $p < 0.05$, $n = 5$ cells). Error bars represent SEM. Scale bar 5 μm.

$p = 0.28$; mean decay time: spontaneous 3.61 s, uncaging 3.11 s, $p = 0.81$). Whole-cell patch clamp electrophysiology further confirmed the presence of miniature NMDA excitatory postsynaptic currents (EPSCs) at this developmental stage (Figure 1E). In order to confirm that similar spontaneous events occur in a more intact environment, we imaged immature dendrites in young hippocampal slices (corresponding to P3–P4) in the presence of TTX to block all evoked release. We identified similar, highly localized, NMDA-dependent, dendritic spontaneous mCaTs (Figure 2).

Spontaneous Dendritic Events Are due to Axonal Vesicular Release

We initially attempted to block these spontaneous Ca^{2+} responses using botulinum toxin A (BoNT-A), which disrupts vesicle release by cleavage of SNAP25 (Blasi et al., 1993). However, we found that, as has been shown for tetanus toxin (Choi et al., 2014; Shin et al., 2012; Verderio et al., 1999), BoNT-A failed to substantially inhibit spontaneous release in young neurons, measured with FM dyes and biosyn, despite successfully preventing evoked release (Figures S2A–S2C). Co-staining of biosyn with vGlut1 confirmed that the lack of effect was not due to inhibitory cell identity (Figure S2C). As an alternative approach, we used the enzyme glutamate pyruvate transaminase (GPT) as a glutamate scavenger, which should intercept glutamate molecules released from neighboring boutons (Figure 3A). Bath application of GPT (20 U/ml + 6 mM pyruvate), which inhibited calcium events driven by glutamate uncaging (Figure 3B), also led to a significant reduction in spontaneous mCaTs compared with a pyruvate only control (GPT: $p < 0.01$, Figure 3C). To further confirm that these mCaTs were due to presynaptic vesicular release, we applied brief puffs of hypertonic sucrose to induce release of the readily releasable pool of vesicles (Rosenmund and Stevens, 1996). This rapidly induced a dramatic transient increase in mCaT frequency in hot spots along the dendrite, including those that were previously active (Figure 3D). Additionally, acute application of the drug Bafilomycin, which we have recently found is able to drive a transient increase in the rate of spontaneous synaptic release in mature neurons (Figure S2D), resulted in a concomitant increase in mCaT frequency at 4–5 DIV, which was blocked by APV (Figures S2E and S2F).

Glutamate Release Can Activate Distant NMDA Receptors

Although the peak phase of synapse formation occurs later in development, from 7–10 DIV in culture, we initially hypothesized that these mCaTs might reflect release from very early, pioneering synapses. We therefore carried out post hoc immunostaining for vGlut1 after recording spontaneous mCaTs at 4–5 DIV to establish if mCaTs colocalized with vGlut1 boutons, the source of released glutamate. To our surprise, we found that only 9% of mCaTs were located adjacent to a vGlut1 punctum (Figure 4A). We therefore quantified the distance from the nearest glutamate release site for all mCaTs and found a distribution that peaked at 5–10 μm but still showed many responses occurring at 15–20 μm (Figure 4B). This compares with a synaptic cleft width of approximately 20 nm in mature synaptic contacts. Given the relatively rapid rate of glutamate diffusion, it seemed very surprising that vesicular glutamate release could drive clear, discrete calcium responses at such long distances in the range of tens of microns. Therefore, to establish whether this was indeed technically possible, we used focal glutamate uncaging to mimic vesicle release and targeted the uncaging laser to varying distances from $GCaMP3^{+}$ dendrites. Critically, we used uncaging pulses (0.5–1 ms) that we know to be equivalent to quantal release of glutamate, as we have previously calibrated our system by matching uncaging pulses to electrophysiologically recorded AMPA-driven miniature excitatory postsynaptic currents (mEPSCs) (Kay et al., 2011). We could measure robust Ca^{2+} responses at up to 20 μm, with a highly distance-dependent response amplitude (Figure 4C). To exclude astrocytic release as a potential source of glutamate, we cultured pure populations

OPEN
ACCESS
CellPress

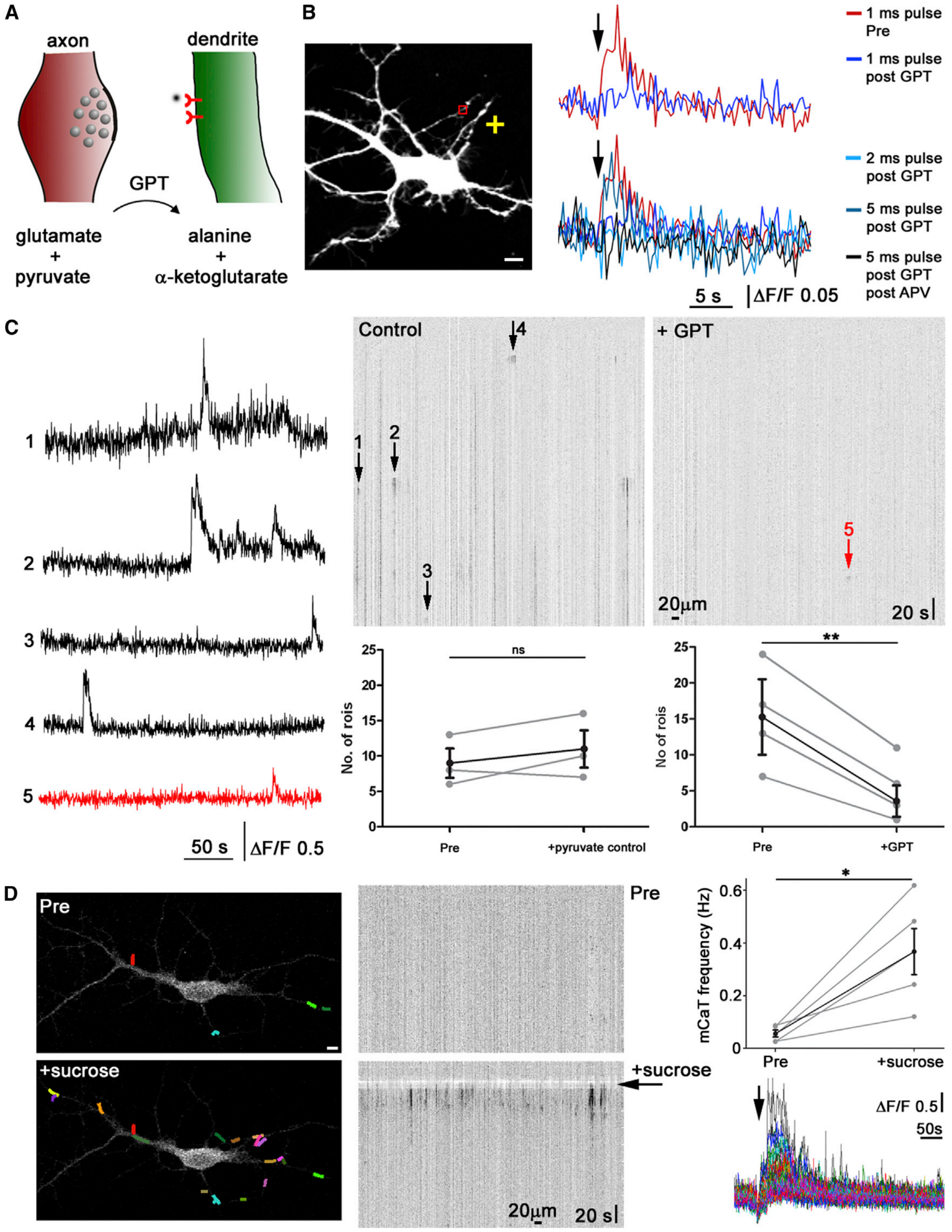

Figure 3. Spontaneous mCaTs Inhibited by Glutamate Scavenger and Triggered by Sucrose-Induced Vesicular Release

(A) Schematic depicting action of the enzyme GPT, which in the presence of pyruvate converts glutamate to alanine and α-ketoglutarate.

(B) Calibration of GPT effect using focal uncaging (+ on image) and fluorescent traces from the region of interest (red box) shown at right. Black arrow indicates time of uncaging. A 1-ms uncaging pulse induces a calcium response that is abolished by 20 U/ml GPT+ 6 mM pyruvate (top traces). Increasing the glutamate pulse to 5 ms, but not 2 ms, can overcome the action of the enzyme while all responses are abolished by the addition of APV (bottom traces).

(legend continued on next page)

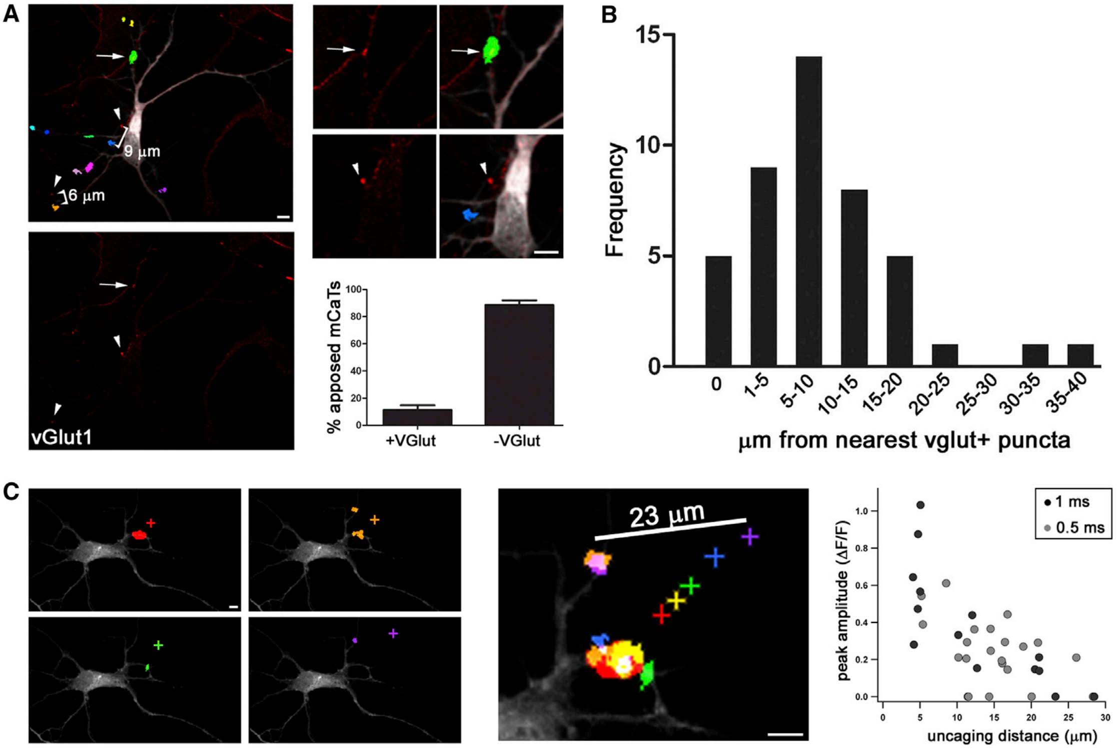

Figure 4. Glutamate Release May Act at Long-Range Early in Development

(A) Post hoc immunostaining with vGlut1 overlaid onto mCaT responses indicates that many response sites are localized at a distance from the nearest glutamate source. (Left) Example neuron with vGlut1 staining only below, and mCaTs identified using automated software and pseudocolored in random colors. The arrows indicate sites of apposed vGlut1 puncta and mCaT response (high magnification: right, top). Arrowheads show vGlut1 puncta located at the distance indicated from mCaT (right, center). (Below) Graph shows percentage of mCaTs that abut a glutamate release site.

(B) Distribution of distances between mCaT response site and nearest vGlut1.

(C) Distance mapping using focal glutamate uncaging. (Left) Individual examples of uncaging responses. (Center) Overlay of all uncaging events (color coded). (Right) Peak ΔF/F as a function of uncaging distance.

Error bars represent SEM. Scale bars are 5 μm.

of neurons above a glial feeder layer (Kaech and Banker, 2006) so that imaging could be conducted in the absence of glia. We confirmed that in these circumstances we continued to see spontaneous mCaTs (data not shown).

Spontaneous Release of Glutamate Affects Dendritic Arbor Complexity

We speculated that the spontaneous release of glutamate acting on dendritic NMDA receptors at an early developmental stage, prior to axodendritic contact and synapse formation, might promote dendritic branching or deviation, consistent with studies suggesting that dendritic filopodia may initiate contact (Ziv and Smith, 1996) and stabilize at specific presynaptic sites (Sabo et al., 2006). To dissociate effects due to evoked release from spontaneous release, we took advantage of the time-locked developmental properties of the dissociated neuronal preparation. Our previous data indicated that at 4 DIV neurons undergo exceptionally high levels of spontaneous vesicle cycling in the absence of evoked release. Spontaneous release is gradually wound down as neurons mature, while activity-dependent release is activated at around 7–8 DIV (Andreae et al., 2012). We therefore used bath application of either TTX to prevent action potential firing or APV to block NMDA-dependent signaling, for 48-hr periods at different developmental stages (Figure 5A). NMDA blockade coinciding with the period of exclusive spontaneous release (1–3 DIV) resulted in a significant reduction in dendritic branching (branch density $p = 0.006$, number of branch points $p = 0.005$, all one-way ANOVA with Dunnett posttests), the number of dendritic processes ($p = 0.006$), and total dendritic length ($p = 0.004$). Further, APV treatment at this stage resulted in a corresponding increase in dendrite straightness ($p = 0.0001$) and more symmetrical arbors ($p = 0.008$). As expected, TTX

(C) Example neuron with kymographs showing spontaneous mCaTs (example traces in black), which are significantly reduced following application of 20 U/ml GPT (+pyruvate) (remaining event in red). Quantification shows the number of responding regions before and after pyruvate control (left, n = 3 cells) or GPT (right, $p < 0.01$, n = 4).

(D) Hypertonic sucrose application (arrow) induces a dramatic increase in mCaT frequency. The example neuron at the left shows mCaT locations in random colors, example kymographs shown in center, quantification of sucrose effect and example mCaT traces (n = 5) at right.

Error bars represent SEM; scale bar is 5 μm. See also Figure S2.

OPEN ACCESS
CellPress

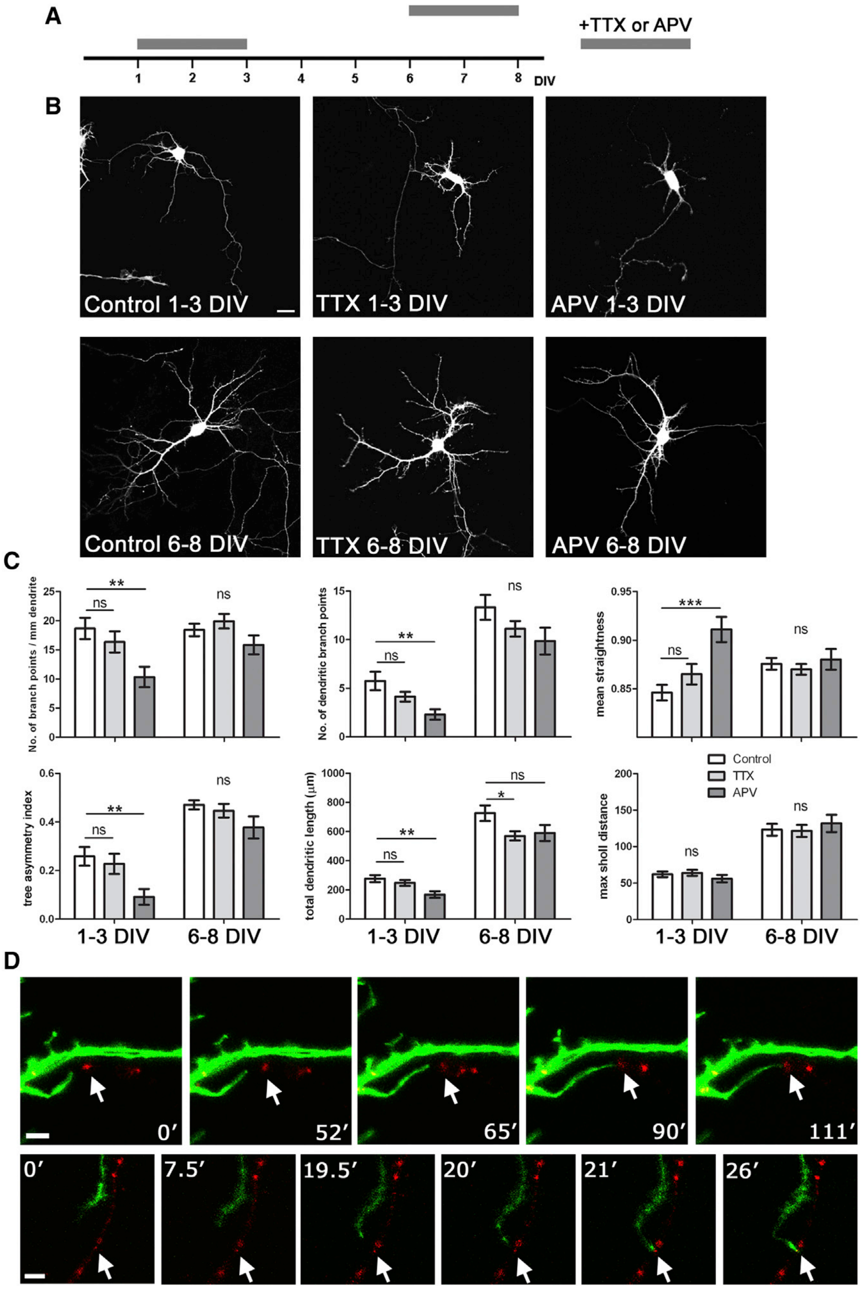

Figure 5. Spontaneous Glutamate Release Directs Dendritic Arbor Complexity

(A) Schematic showing TTX or APV treatment timelines.

(B) Representative images of neurons transfected with GFP and treated with TTX or APV at the times shown; scale bar represents 20 μm.

(C) Quantification of dendritic arbor parameters: branch point density, number of branch points, dendritic straightness, tree asymmetry index (where 0 = symmetrical, 1 = asymmetrical), total dendritic length, and maximal sholl distance in control (white, n ≥ 21 cells), TTX-treated (light gray, n ≥ 23 cells), and APV-treated (dark gray, n ≥ 20 cells) neurons.

(D) Dual-color imaging of two neurons, dendrites of one expressing GFP (green), and axonal synaptic vesicle clusters expressing tagged VAMP2 (red). Stills from movies with time in minutes indicated; scale bar represents 5 μm. Arrow indicates "target" vesicle cluster.

Error bars represent SEM. See also Figure S3.

had no significant effects at this early stage. However, by 6–8 DIV, almost no significant effects due to APV were seen in any dendritic measure, although TTX treatment now resulted in a reduction in total dendritic length (Figures 5B, 5C, and S3). These results indicate a significant reduction in dendritic complexity and, together with the increased dendritic straightness and more symmetrical arborizations, are consistent with the loss of a deviating or branching cue. To attempt to capture live the local interaction between axonal release site and dendrite prior to contact, we imaged independently transfected neurons expressing either GFP in dendrites or biosyn at sites of spontaneous vesicle cycling. We were able to obtain two examples where a dendritic process appeared to extend toward and target an axonal release site (Figure 5D), suggestive of a possible guidance role.

DISCUSSION

We find that early in neuronal development the spontaneous vesicular release of the neurotransmitter glutamate can be sensed by distant NMDA receptors localized to discrete regions of developing dendrites. Interfering with this signal leads to a reduction in dendritic arbor complexity and points toward a role for spontaneous glutamate release prior to synapse formation to promote or perhaps even guide local dendritic branches.

Early Glutamate Release Is Vesicular in Nature

Axonal release of the neurotransmitter acetylcholine prior to synapse formation has been previously described (Hume et al., 1983; Young and Poo, 1983) and interpreted to be due to vesicle fusion events at sites reminiscent of presynaptic boutons (Zakharenko et al., 1999). We believe that all of the evidence presented here strongly points to glutamate originating from presynaptic vesicular release events. First, all cycling vesicles are labeled via known presynaptic vesicle proteins such as VAMP2 and synaptophysin. The spontaneous dendritic calcium responses we record show similar amplitudes and kinetics to focal uncaging pulses that have been matched to single vesicle release events. Although previous studies have interpreted a lack of effect from botulinum toxin as indicating that release may be nonvesicular, we find that early in development

spontaneous vesicular release is resistant to treatment with BoNT-A, as has previously been described for tetanus toxin (Shin et al., 2012; Verderio et al., 1999). This represents an important finding with implications not only for how experiments that use this toxin are interpreted, but potentially relevant to our understanding of how this vesicle pool operates at a molecular level. In this study, we have focused on the effects of this early, high-level spontaneous release on neighboring dendrites, but it will be important in the future to characterize the molecular identity of this vesicle pool. A potential candidate would be the vSNARE Vti1a, which has recently been identified as involved in spontaneous vesicle cycling in mature neurons (Ramirez et al., 2012). Critically, we have used a well-established method (hypertonic sucrose) to trigger vesicular release from mature presynaptic boutons (Rosenmund and Stevens, 1996), which resulted in a concomitant increase in dendritic mCaTs. Additionally, we have identified a novel action for the drug Bafilomycin, which can induce a transient increase in spontaneous vesicular release in both mature synapses (resulting in a rapid increase in mEPSC frequency) and in young neurons. This not only adds weight to our interpretation that the dendritic events are due to axonal vesicular release, but also has implications for the use of this drug when studying vesicle cycling in presynaptic terminals.

Spontaneous Release of Glutamate Is a Regulator of Dendritic Development

Presynaptic glutamate release has long been known to have effects on dendritic spines. For example, high-frequency glutamate release in mature neurons (via electrical stimulation or focal uncaging) can induce both filopodia and spine formation (Engert and Bonhoeffer, 1999; Kwon and Sabatini, 2011; Maletic-Savatic et al., 1999). Glutamate application can induce filopodia formation in cultured neurons (Cornell-Bell et al., 1990) and increased filopodial length (Portera-Cailliau et al., 2003), although the latter effect may be mediated by metabotropic glutamate receptors (Cruz-Martín et al., 2012). Equally, ionotropic glutamate receptor blockade inhibits dendritic filopodial motility in retinal ganglion cells (Wong and Wong, 2001). There is also evidence that in *Xenopus* tectal neurons dendritic arbor growth is regulated by glutamate-dependent activity, including effects mediated via NMDA receptors (Rajan and Cline, 1998; Rajan et al., 1999; Sin et al., 2002). Previous studies have found that NMDA receptor blockade restricts dendritic development (Ewald et al., 2008; Lee et al., 2005), although it is also reported that NMDA antagonists may promote branching (Lin and Constantine-Paton, 1998; McAllister et al., 1996). This discrepancy may partially be explained by evidence suggesting that glutamate receptor activation/blockade can have different effects at different developmental stages (Tashiro et al., 2003; Wong and Wong, 2001). Interestingly, mice exhibiting hypofunction of NMDA receptors (due to deletion of the enzyme that generates the NMDA receptor cofactor, serine) show significant reductions in dendritic complexity in the somatosensory cortex (Balu et al., 2012), and deletion of the developmentally expressed NMDA receptor subunit GluN2B from the CA3 region of the hippocampus results in a reduction in the density of dendritic spines (Akashi et al., 2009). Tantalizingly, dendritic filopodia appear to stabilize at synaptic vesicle pause sites (Sabo et al., 2006). Although it has long been hypothesized that glutamate may indeed attract dendrites, to our knowledge, this has never been directly observed. Our results now offer a new perspective on these previous studies by specifically identifying spontaneous release as the key form of neurotransmission involved in regulating NMDA-dependent dendritic arbor growth and complexity prior to synapse formation, potentially by releasing glutamate to act as a local branching or guidance cue. Our work adds a new, critical role for spontaneous neurotransmission during development, giving further weight to the view that this form of release is not merely "noise" but an important and independent phenomenon (Ramirez and Kavalali, 2011).

NMDA Receptors—A Coincidence Detector in Development?

Classically, NMDA receptors operate as coincidence detectors to gate synaptic plasticity in situations where neuronal depolarization has relieved the magnesium block, allowing the channel to open in response to glutamate. It is possible that glutamate binding to NMDA receptors in our system could be acting via non-ionotropic actions that do not require depolarization and relief of magnesium block (Vissel et al., 2001). Alternatively, where glutamate release coincides with dendritic depolarization, this could allow NMDA receptor signaling which in turn promotes dendritic growth. Spontaneous waves of depolarization have been well described in the developing hippocampus (and, indeed, cortex) which could perform this function (Allène et al., 2008; Crépel et al., 2007).

This study has primarily used dissociated neurons in order to dissect out the specific role of spontaneous neurotransmission at this stage. However, the data would fit well with the existing literature in terms of the developmental timing of such a role. Excitatory pyramidal neurons in the rodent hippocampus are known to undergo a stereotypic pattern of development where GABAergic inputs (still depolarizing at this stage) precede glutamatergic inputs (Tyzio et al., 1999) and can potentiate NMDA receptor responses (Leinekugel et al., 1997). The dendritic arbor is short and simple when neurons exhibit GABAergic inputs only, but has increased significantly in complexity by the time glutamatergic synapses have formed onto the neurons (Tyzio et al., 1999). Thus, the environment is ideal for regular depolarization of the developing neuron to coincide with glutamate release from incoming axons, allowing NMDA receptor activation and downstream effects on dendritic growth. Interestingly, blockade of GABA-dependent depolarization has been shown to impair excitatory synapse formation and cause a reduction in dendritic branching and spine density in cortical pyramidal neurons (Wang and Kriegstein, 2008, 2011). The effects on excitatory synapse formation could be rescued by early (but not late) expression of a mutant NMDA receptor that lacks the normal voltage-dependent channel block, supporting the idea that the combination of depolarizing GABA and NMDA receptor activation is critical for excitatory synapse formation (Wang and Kriegstein, 2008).

Long-Distance Effects

We have used a culture system to provide a proof of principle that glutamate could signal to surprisingly distant NMDA

receptors early in development. Could such a process occur in vivo? Although we do not anticipate that the actual distances would necessarily be as extreme as in vitro, there is no reason to believe that glutamate could not act as a short-range guidance cue (reviewed in Andreae and Burrone, 2014). For example, there is a great deal of evidence that the rapid uptake of glutamate that occurs in adult neuronal tissue is profoundly slowed early in development due to a delay in transporter expression (Thomas et al., 2011). Using optical sensors of glutamate in the developing retina, a recent study directly demonstrated high levels of extrasynaptic glutamate secondary to activity (Firl et al., 2013). At the same time, young neurons should be better able to detect low levels of glutamate as they express the more sensitive NMDA receptor subunit, NR2B (Laurie and Seeburg, 1994). Indeed, studies of glutamate "spillover" in the cerebellum have demonstrated diffusion of glutamate for distances in the tens of microns in intact tissue (Carter and Regehr, 2000). Together, these findings suggest that the developing brain is a permissive environment for long-rage communication and that neurotransmitters released by axons may serve to fine-tune connectivity between neurons.

EXPERIMENTAL PROCEDURES

Animals

All animal procedures were approved by the local ethics committee and the UK Home Office.

In Utero Virus Injection

AAV1.hSyn.GCaMP5G.WPRE.SV40 (University of Pennsylvania) at 1.2×10^{10} gc/μl was mixed with 0.005% Fast Green and injected into the lateral ventricle of E13.5 C57BL/6 mouse embryos.

Hippocampal Organotypic Slices and Dissociated Cultures

A dorsoposterior region containing the hippocampus was dissected from brains of P1 mice and 300 μm organotypic slices cultured on Millicell membranes at a gas/liquid interface (Stoppini et al., 1991). Primary hippocampal cultures were prepared from embryonic day 18 Sprague-Dawley rats. Hippocampi were dissociated and plated at 350 cells/mm^2 on glass coverslips coated with poly-D-lysine (50 μg/ml) and laminin (20 μg/ml). After 3–6 DIV, neurons were transfected using Effectene (Invitrogen).

Calcium Imaging and Uncaging

Organotypic slices were transferred to an imaging chamber and superfused with oxygenated (95% O_2/5% CO_2) artificial cerebrospinal fluid (ACSF; composition in mM: NaCl 119, KCl 2.5, $CaCl_2$ 2, $MgCl_2$ 0, NaH_2PO_4 1, $NaHCO_3$ 26.2, glucose 11, serine 0.01, Trolox 2.2) at 32°C. Dissociated neurons transfected with GCaMP3 at 3 DIV were transferred into HEPES-buffered saline (HBS) containing (in mM): 139 NaCl, 2.5 KCl, 10 HEPES, 10 D-glucose, 2 $CaCl_2$ with 0 Mg^{2+}, and 1 μM TTX at 4–5 DIV. Time-lapse images were acquired at ~3 Hz on a FV1000 Olympus confocal microscope. Caged MNI-glutamate (Tocris, Bristol, UK) was added to the bath at 0.5 mM and uncaging performed using a 405 nm laser (4.7 mW, pulses between 0.5 and 1 ms). For hypertonic sucrose experiments, 0.5 M sucrose in HBS with 0 Mg^{2+} and 1 μM TTX was puffed over the neuron while acquiring time-lapse images. For glutamate scavenging studies, glutamate pyruvate dehydrogenase (GPT, Sigma) at 20 U/ml was co-applied to the bath with 6 mM pyruvate (Sigma). Bafilomycin (Calbiochem) was bath applied at 1.6 mM.

Labeling Vesicles with Biosyn, SynaptopHysin-pHluorin, FM4-64, and Immunocytochemistry

Synaptic vesicles were labeled with Biosyn or synaptopHysin-pHluorin as previously described (Andreae et al., 2012). BoNT-A (Metabiologics) was bath applied to culture media at 100 ng/ml for 48 hr. For FM dye labeling, neurons were incubated in 10 μM FM4-64 in culture media still containing BoNT-A for 15 min at 37°C. Neurons were depolarized with 40 APs at 20 Hz. For antibody staining, neurons were permeabilized with 0.1% saponin, blocked with 3% BSA, and incubated in primary antibody followed by Alexa-dye conjugated secondary antibody. Primary antibodies used were vGlut1, vGlut2 (Synaptic Systems), and GFP (Millipore). Images were obtained using a FV1000 Olympus confocal microscope. Colocalization analysis was done with automated custom written routines in MATLAB.

Electrophysiology

Whole-cell patch-clamp recordings of mEPSCs were recorded (HEKA EPC10/2 amplifier, Pulse software) from dissociated neurons. Electrode tips with a resistance of 2–5 MΩ (mature neurons) or 4–7 MΩ (young neurons) were filled with an intracellular solution containing (in mM): 130 K^+-gluconate, 10 NaCl, 1 EGTA, 0.133 $CaCl_2$, 2 $MgCl_2$ (AMPA mEPSCs) or 0 $MgCl_2$ (NMDA mEPSCs), 10 HEPES, 3.5 NaATP, 1 NaGTP. Neurons were clamped at −70 mV for AMPA mEPSC recordings in mature neurons and at resting membrane potential (Vm ~−40 mV with junction potential correction) for NMDA mEPSCs in young neurons. Cells were maintained in HBS plus 1 μM TTX and 10 μM gabazine to record AMPA mEPSCs in mature neurons and in modified HBS with nominally 0 mM $MgCl_2$, plus 1 μM TTX, 20 μM CNQX, and 10 μM gabazine to isolate NMDA mEPSCs in young neurons.

Data Analysis and Statistics

Images were analyzed using custom-written routines in MATLAB (MathWorks). Time-lapse movies (calcium imaging) in slices were realigned where necessary in ImageJ using cvMatch_Template plugin (Q Tseng). Dendrites were traced in NeuronJ (Meijering et al., 2004) and then imported into MATLAB. The single-pixel trace was then dilated to three pixels thick and the mean normalized fluorescence (ΔF/F) over the three pixels calculated over the entire time series. The data were presented as a kymograph where the x axis represents the concatenation of all dendrites into a single line and the y axis represents time (s). Episodes of increased fluorescence are visible as dark spots on the kymograph. Positive mCaTs were identified using a two-stage process: initially, sections of dendrites which appeared to respond were picked by hand by an experimenter blind to treatment group; subsequently, the 2D traces were processed in Minianalysis (Synaptosoft) and true positives confirmed on an automated basis where the threshold was set at 4*SD of baseline. In some cases, data were also analyzed using entirely automated software (IVFA) on an unbiased pixel-by-pixel basis (Nikolaou et al., 2012), as seen in Figure 4. For SypHy data, puncta were automatically picked using edge detection on thresholded images in MATLAB and average normalized fluorescence for each region of interest calculated over time. Analysis of dendritic arborization was performed in density-matched cultured neurons blind to experimental group, with dendritic tree tracings of sparsely transfected GFP-expressing neurons done in NeuronJ and automated analysis by custom-written MATLAB routines (Matthew Grubb) based on work by Uylings and Pelt, which generates multiple parameters that are better suited to analysis of immature dendritic trees than only a simple Sholl analysis (Uylings and van Pelt, 2002). Miniature EPSCs were analyzed blind to experimental group and without manual editing using Minianalysis software. Statistical analysis was carried out in Prism (GraphPad) or MATLAB using, where appropriate, paired or unpaired Student's t test for two groups or one-way ANOVA for three groups with post hoc Tukey's multiple comparison test.

SUPPLEMENTAL INFORMATION

Supplemental Information includes three figures and can be found with this article online at http://dx.doi.org/10.1016/j.celrep.2015.01.032.

AUTHOR CONTRIBUTIONS

L.C.A. performed the experiments. L.C.A. and J.B. designed the experiments, analyzed the data, and wrote the manuscript.

ACKNOWLEDGMENTS

This work was supported by the Medical Research Council, the Wellcome Trust, the European Research Council, and a Lister prize fellowship to J.B. We thank Mideia Kotsogianni and Gabriel Nadeau for technical help, Andrew Lowe for use of the IVFA software, and Matthew Grubb and Martin Meyer for critical reading of the manuscript.

Received: October 7, 2014
Revised: December 19, 2014
Accepted: January 10, 2015
Published: February 12, 2015

REFERENCES

Akashi, K., Kakizaki, T., Kamiya, H., Fukaya, M., Yamasaki, M., Abe, M., Natsume, R., Watanabe, M., and Sakimura, K. (2009). NMDA receptor GluN2B (GluR epsilon 2/NR2B) subunit is crucial for channel function, postsynaptic macromolecular organization, and actin cytoskeleton at hippocampal CA3 synapses. J. Neurosci. *29*, 10869–10882.

Allène, C., Cattani, A., Ackman, J.B., Bonifazi, P., Aniksztejn, L., Ben-Ari, Y., and Cossart, R. (2008). Sequential generation of two distinct synapse-driven network patterns in developing neocortex. J. Neurosci. *28*, 12851–12863.

Andreae, L.C., and Burrone, J. (2014). The role of neuronal activity and transmitter release on synapse formation. Curr. Opin. Neurobiol. *27*, 47–52.

Andreae, L.C., Fredj, N.B., and Burrone, J. (2012). Independent vesicle pools underlie different modes of release during neuronal development. J. Neurosci. *32*, 1867–1874.

Balu, D.T., Basu, A.C., Corradi, J.P., Cacace, A.M., and Coyle, J.T. (2012). The NMDA receptor co-agonists, D-serine and glycine, regulate neuronal dendritic architecture in the somatosensory cortex. Neurobiol. Dis. *45*, 671–682.

Basarsky, T.A., Parpura, V., and Haydon, P.G. (1994). Hippocampal synaptogenesis in cell culture: developmental time course of synapse formation, calcium influx, and synaptic protein distribution. J. Neurosci. *14*, 6402–6411.

Blasi, J., Chapman, E.R., Link, E., Binz, T., Yamasaki, S., De Camilli, P., Südhof, T.C., Niemann, H., and Jahn, R. (1993). Botulinum neurotoxin A selectively cleaves the synaptic protein SNAP-25. Nature *365*, 160–163.

Bleckert, A., and Wong, R.O. (2011). Identifying roles for neurotransmission in circuit assembly: insights gained from multiple model systems and experimental approaches. BioEssays *33*, 61–72.

Carter, A.G., and Regehr, W.G. (2000). Prolonged synaptic currents and glutamate spillover at the parallel fiber to stellate cell synapse. J. Neurosci. *20*, 4423–4434.

Choi, B.J., Imlach, W.L., Jiao, W., Wolfram, V., Wu, Y., Grbic, M., Cela, C., Baines, R.A., Nitabach, M.N., and McCabe, B.D. (2014). Miniature neurotransmission regulates Drosophila synaptic structural maturation. Neuron *82*, 618–634.

Chung, C., Barylko, B., Leitz, J., Liu, X., and Kavalali, E.T. (2010). Acute dynamin inhibition dissects synaptic vesicle recycling pathways that drive spontaneous and evoked neurotransmission. J. Neurosci. *30*, 1363–1376.

Cline, H., and Haas, K. (2008). The regulation of dendritic arbor development and plasticity by glutamatergic synaptic input: a review of the synaptotrophic hypothesis. J. Physiol. *586*, 1509–1517.

Cornell-Bell, A.H., Thomas, P.G., and Smith, S.J. (1990). The excitatory neurotransmitter glutamate causes filopodia formation in cultured hippocampal astrocytes. Glia *3*, 322–334.

Crépel, V., Aronov, D., Jorquera, I., Represa, A., Ben-Ari, Y., and Cossart, R. (2007). A parturition-associated nonsynaptic coherent activity pattern in the developing hippocampus. Neuron *54*, 105–120.

Cruz-Martín, A., Crespo, M., and Portera-Cailliau, C. (2012). Glutamate induces the elongation of early dendritic protrusions via mGluRs in wild type mice, but not in fragile X mice. PLoS ONE *7*, e32446.

Dailey, M.E., and Smith, S.J. (1996). The dynamics of dendritic structure in developing hippocampal slices. J. Neurosci. *16*, 2983–2994.

Engert, F., and Bonhoeffer, T. (1999). Dendritic spine changes associated with hippocampal long-term synaptic plasticity. Nature *399*, 66–70.

Ewald, R.C., Van Keuren-Jensen, K.R., Aizenman, C.D., and Cline, H.T. (2008). Roles of NR2A and NR2B in the development of dendritic arbor morphology in vivo. J. Neurosci. *28*, 850–861.

Firl, A., Sack, G.S., Newman, Z.L., Tani, H., and Feller, M.B. (2013). Extrasynaptic glutamate and inhibitory neurotransmission modulate ganglion cell participation during glutamatergic retinal waves. J. Neurophysiol. *109*, 1969–1978.

Frank, C.A., Kennedy, M.J., Goold, C.P., Marek, K.W., and Davis, G.W. (2006). Mechanisms underlying the rapid induction and sustained expression of synaptic homeostasis. Neuron *52*, 663–677.

Fredj, N.B., and Burrone, J. (2009). A resting pool of vesicles is responsible for spontaneous vesicle fusion at the synapse. Nat. Neurosci. *12*, 751–758.

Hume, R.I., Role, L.W., and Fischbach, G.D. (1983). Acetylcholine release from growth cones detected with patches of acetylcholine receptor-rich membranes. Nature *305*, 632–634.

Kaech, S., and Banker, G. (2006). Culturing hippocampal neurons. Nat. Protoc. *1*, 2406–2415.

Kay, L., Humphreys, L., Eickholt, B.J., and Burrone, J. (2011). Neuronal activity drives matching of pre- and postsynaptic function during synapse maturation. Nat. Neurosci. *14*, 688–690.

Kwon, H.B., and Sabatini, B.L. (2011). Glutamate induces de novo growth of functional spines in developing cortex. Nature *474*, 100–104.

Laurie, D.J., and Seeburg, P.H. (1994). Ligand affinities at recombinant N-methyl-D-aspartate receptors depend on subunit composition. Eur. J. Pharmacol. *268*, 335–345.

Lee, L.J., Lo, F.S., and Erzurumlu, R.S. (2005). NMDA receptor-dependent regulation of axonal and dendritic branching. J. Neurosci. *25*, 2304–2311.

Lee, M.C., Yasuda, R., and Ehlers, M.D. (2010). Metaplasticity at single glutamatergic synapses. Neuron *66*, 859–870.

Leinekugel, X., Medina, I., Khalilov, I., Ben-Ari, Y., and Khazipov, R. (1997). Ca2+ oscillations mediated by the synergistic excitatory actions of GABA(A) and NMDA receptors in the neonatal hippocampus. Neuron *18*, 243–255.

Lin, S.Y., and Constantine-Paton, M. (1998). Suppression of sprouting: An early function of NMDA receptors in the absence of AMPA/kainate receptor activity. J. Neurosci. *18*, 3725–3737.

Lohmann, C., and Bonhoeffer, T. (2008). A role for local calcium signaling in rapid synaptic partner selection by dendritic filopodia. Neuron *59*, 253–260.

Maletic-Savatic, M., Malinow, R., and Svoboda, K. (1999). Rapid dendritic morphogenesis in CA1 hippocampal dendrites induced by synaptic activity. Science *283*, 1923–1927.

McAllister, A.K., Katz, L.C., and Lo, D.C. (1996). Neurotrophin regulation of cortical dendritic growth requires activity. Neuron *17*, 1057–1064.

McKinney, R.A., Capogna, M., Dürr, R., Gähwiler, B.H., and Thompson, S.M. (1999). Miniature synaptic events maintain dendritic spines via AMPA receptor activation. Nat. Neurosci. *2*, 44–49.

Meijering, E., Jacob, M., Sarria, J.C., Steiner, P., Hirling, H., and Unser, M. (2004). Design and validation of a tool for neurite tracing and analysis in fluorescence microscopy images. Cytometry Part A *58*, 167–176.

Niell, C.M., Meyer, M.P., and Smith, S.J. (2004). In vivo imaging of synapse formation on a growing dendritic arbor. Nat. Neurosci. *7*, 254–260.

Nikolaou, N., Lowe, A.S., Walker, A.S., Abbas, F., Hunter, P.R., Thompson, I.D., and Meyer, M.P. (2012). Parametric functional maps of visual inputs to the tectum. Neuron *76*, 317–324.

Portera-Cailliau, C., Pan, D.T., and Yuste, R. (2003). Activity-regulated dynamic behavior of early dendritic protrusions: evidence for different types of dendritic filopodia. J. Neurosci. *23*, 7129–7142.

Rajan, I., and Cline, H.T. (1998). Glutamate receptor activity is required for normal development of tectal cell dendrites in vivo. J. Neurosci. *18*, 7836–7846.

Rajan, I., Witte, S., and Cline, H.T. (1999). NMDA receptor activity stabilizes presynaptic retinotectal axons and postsynaptic optic tectal cell dendrites in vivo. J. Neurobiol. *38*, 357–368.

Ramirez, D.M., and Kavalali, E.T. (2011). Differential regulation of spontaneous and evoked neurotransmitter release at central synapses. Curr. Opin. Neurobiol. *21*, 275–282.

Ramirez, D.M., Khvotchev, M., Trauterman, B., and Kavalali, E.T. (2012). Vti1a identifies a vesicle pool that preferentially recycles at rest and maintains spontaneous neurotransmission. Neuron *73*, 121–134.

Renger, J.J., Egles, C., and Liu, G. (2001). A developmental switch in neurotransmitter flux enhances synaptic efficacy by affecting AMPA receptor activation. Neuron *29*, 469–484.

Rosenmund, C., and Stevens, C.F. (1996). Definition of the readily releasable pool of vesicles at hippocampal synapses. Neuron *16*, 1197–1207.

Sabo, S.L., Gomes, R.A., and McAllister, A.K. (2006). Formation of presynaptic terminals at predefined sites along axons. J. Neurosci. *26*, 10813–10825.

Sara, Y., Virmani, T., Deák, F., Liu, X., and Kavalali, E.T. (2005). An isolated pool of vesicles recycles at rest and drives spontaneous neurotransmission. Neuron *45*, 563–573.

Shin, M.C., Nonaka, K., Wakita, M., Yamaga, T., Torii, Y., Harakawa, T., Ginnaga, A., Ito, Y., and Akaike, N. (2012). Effects of tetanus toxin on spontaneous and evoked transmitter release at inhibitory and excitatory synapses in the rat SDCN neurons. Toxicon *59*, 385–392.

Sin, W.C., Haas, K., Ruthazer, E.S., and Cline, H.T. (2002). Dendrite growth increased by visual activity requires NMDA receptor and Rho GTPases. Nature *419*, 475–480.

Stoppini, L., Buchs, P.A., and Muller, D. (1991). A simple method for organotypic cultures of nervous tissue. J. Neurosci. Methods *37*, 173–182.

Sutton, M.A., Ito, H.T., Cressy, P., Kempf, C., Woo, J.C., and Schuman, E.M. (2006). Miniature neurotransmission stabilizes synaptic function via tonic suppression of local dendritic protein synthesis. Cell *125*, 785–799.

Tashiro, A., Dunaevsky, A., Blazeski, R., Mason, C.A., and Yuste, R. (2003). Bidirectional regulation of hippocampal mossy fiber filopodial motility by kainate receptors: a two-step model of synaptogenesis. Neuron *38*, 773–784.

Thomas, C.G., Tian, H., and Diamond, J.S. (2011). The relative roles of diffusion and uptake in clearing synaptically released glutamate change during early postnatal development. J. Neurosci. *31*, 4743–4754.

Tyzio, R., Represa, A., Jorquera, I., Ben-Ari, Y., Gozlan, H., and Aniksztejn, L. (1999). The establishment of GABAergic and glutamatergic synapses on CA1 pyramidal neurons is sequential and correlates with the development of the apical dendrite. J. Neurosci. *19*, 10372–10382.

Uylings, H.B., and van Pelt, J. (2002). Measures for quantifying dendritic arborizations. Network *13*, 397–414.

Vaughn, J.E. (1989). Fine structure of synaptogenesis in the vertebrate central nervous system. Synapse *3*, 255–285.

Verderio, C., Coco, S., Bacci, A., Rossetto, O., De Camilli, P., Montecucco, C., and Matteoli, M. (1999). Tetanus toxin blocks the exocytosis of synaptic vesicles clustered at synapses but not of synaptic vesicles in isolated axons. J. Neurosci. *19*, 6723–6732.

Verhage, M., Maia, A.S., Plomp, J.J., Brussaard, A.B., Heeroma, J.H., Vermeer, H., Toonen, R.F., Hammer, R.E., van den Berg, T.K., Missler, M., et al. (2000). Synaptic assembly of the brain in the absence of neurotransmitter secretion. Science *287*, 864–869.

Vissel, B., Krupp, J.J., Heinemann, S.F., and Westbrook, G.L. (2001). A use-dependent tyrosine dephosphorylation of NMDA receptors is independent of ion flux. Nat. Neurosci. *4*, 587–596.

Wang, D.D., and Kriegstein, A.R. (2008). GABA regulates excitatory synapse formation in the neocortex via NMDA receptor activation. J. Neurosci. *28*, 5547–5558.

Wang, D.D., and Kriegstein, A.R. (2011). Blocking early GABA depolarization with bumetanide results in permanent alterations in cortical circuits and sensorimotor gating deficits. Cereb. Cortex *21*, 574–587.

Washbourne, P., Liu, X.B., Jones, E.G., and McAllister, A.K. (2004). Cycling of NMDA receptors during trafficking in neurons before synapse formation. J. Neurosci. *24*, 8253–8264.

Wong, W.T., and Wong, R.O. (2001). Changing specificity of neurotransmitter regulation of rapid dendritic remodeling during synaptogenesis. Nat. Neurosci. *4*, 351–352.

Wu, G., Malinow, R., and Cline, H.T. (1996). Maturation of a central glutamatergic synapse. Science *274*, 972–976.

Young, S.H., and Poo, M.M. (1983). Spontaneous release of transmitter from growth cones of embryonic neurones. Nature *305*, 634–637.

Zakharenko, S., Chang, S., O'Donoghue, M., and Popov, S.V. (1999). Neurotransmitter secretion along growing nerve processes: comparison with synaptic vesicle exocytosis. J. Cell Biol. *144*, 507–518.

Ziv, N.E., and Smith, S.J. (1996). Evidence for a role of dendritic filopodia in synaptogenesis and spine formation. Neuron *17*, 91–102.

Cell Reports

Supplemental Information

Spontaneous Neurotransmitter Release Shapes Dendritic Arbors via Long-Range Activation of NMDA Receptors

Laura C. Andreae and Juan Burrone

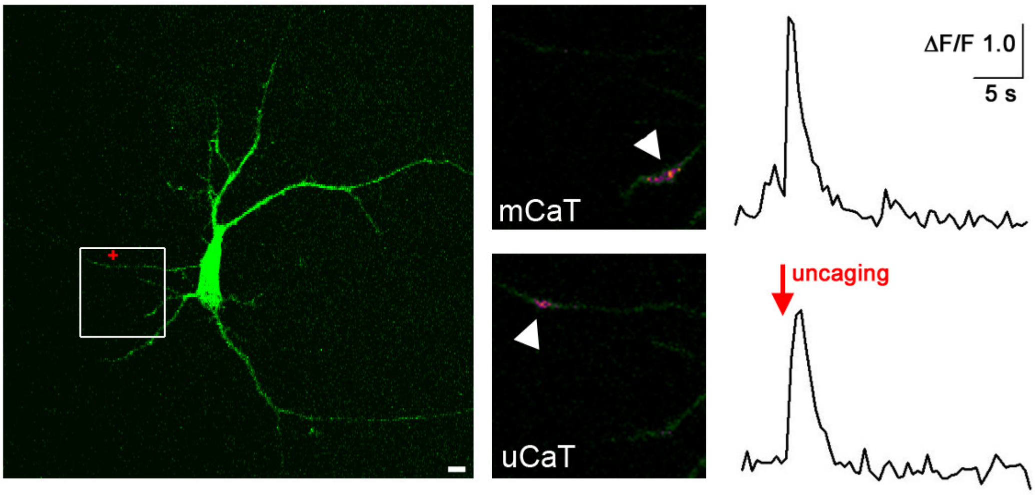

Figure S1, related to Figure 1. Spontaneous and uncaging-induced calcium events in immature dendrites of the same 4 DIV neuron. Left: GCaMP3-expressing neuron, uncaging site indicated (+). Middle panels show high-power views of boxed area, arrowheads indicate response sites, top: example spontaneous mCaT, bottom: uncaging-induced calcium transient (uCaT), corresponding ΔF/F traces to right. Scale bar 5 μm.

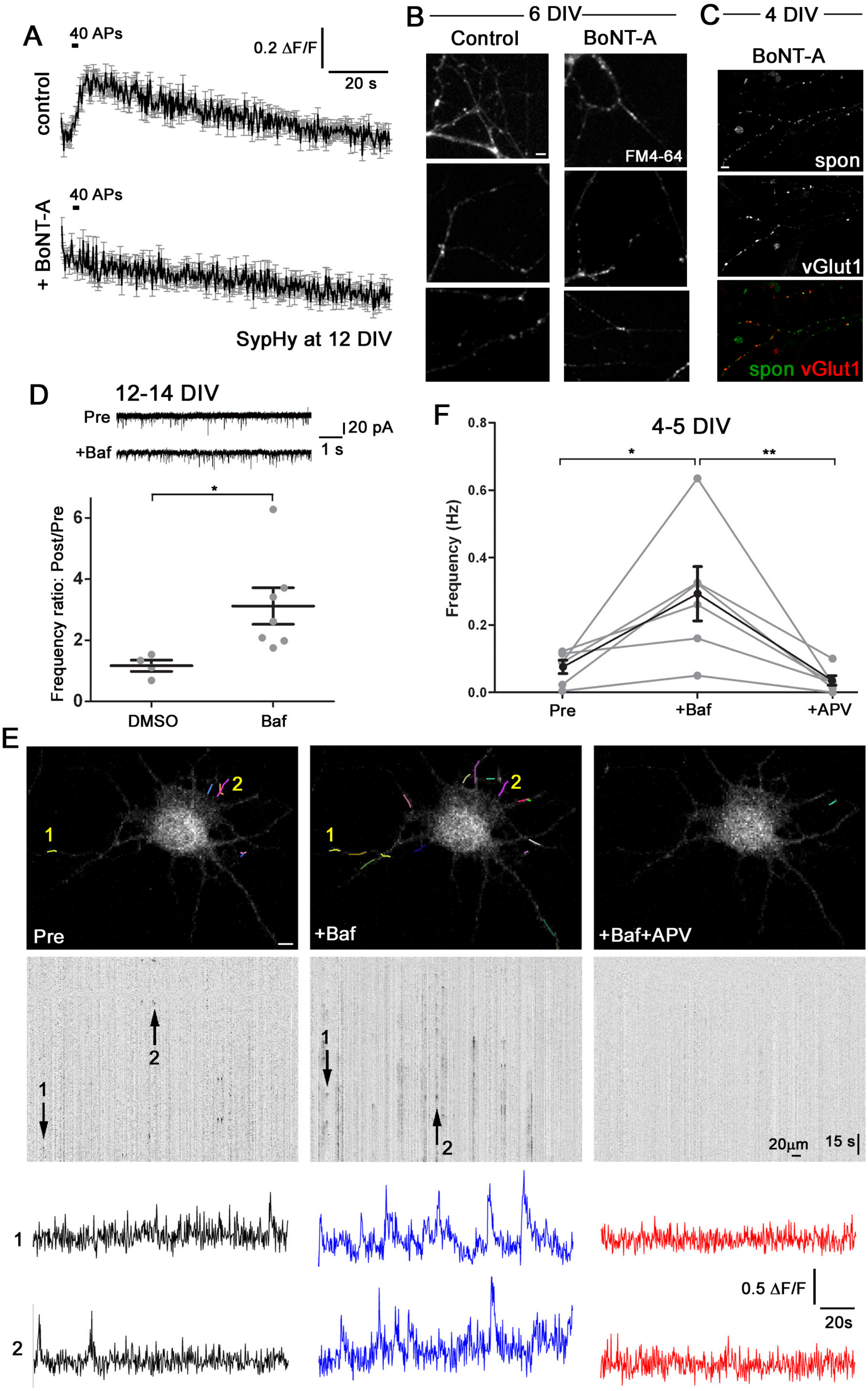
A
40 APs
0.2 ΔF/F
20 s
control
+ BoNT-A
40 APs
SypHy at 12 DIV
B
6 DIV
Control
BoNT-A
FM4-64
C
4 DIV
BoNT-A
spon
vGlut1
spon vGlut1
D
12-14 DIV
Pre
+Baf
20 pA
1 s
Frequency ratio: Post/Pre
DMSO
Baf
F
4-5 DIV
Frequency (Hz)
Pre
+Baf
+APV
E
Pre
+Baf
+Baf+APV
20µm
15 s
0.5 ΔF/F
20s

Figure S2, related to Figure 3. (**A**) In mature (12 DIV) neurons expressing the vesicle cycling reporter SypHy, incubation in 100 ng/ml Botulinum toxin A (BoNT-A) for 48 hrs leads to abolition of the SypHy response to field stimulation of 40 APs at 20Hz, confirming expected activity of the toxin. (**B**) However, similar incubation in BoNT-A has no obvious effect on spontaneous vesicle cycling at 6 DIV, labelled using the styryl dye FM-4-64: 3 representative images for each condition are shown, all taken using identical settings. Similar data was obtained at 3 DIV (not shown). (**C**) Example neuron at 4 DIV expressing biosyn after incubation in BoNT shows robust labelling of spontaneously cycling vesicles (spon); neuron shown is excitatory as indicated by colabelling with vGlut1, so lack of effect from BoNT-A is not due to inhibitory cell identity. (**D**) Electrophysiological recordings in mature neurons demonstrate a significant increase in mEPSC frequency following acute bath application of 1.6 mM Bafilomycin. (**E**) Example neuron at 5 DIV. Top panels: all spontaneous mCaTs shown in random colours, middle panels: corresponding kymograph with 2 examples indicated by arrows, lower panels: $\Delta F/F$ traces for examples): From left to right: baseline spontaneous activity, following bafilomycin application, after subsequent APV (same example ROIs throughout). (**F**) Quantification confirms a significant increase in mCaT frequency following Bafilomycin which is abolished by addition of APV at 4-5 DIV. Error bars represent sem. Scale bars 5 µm.

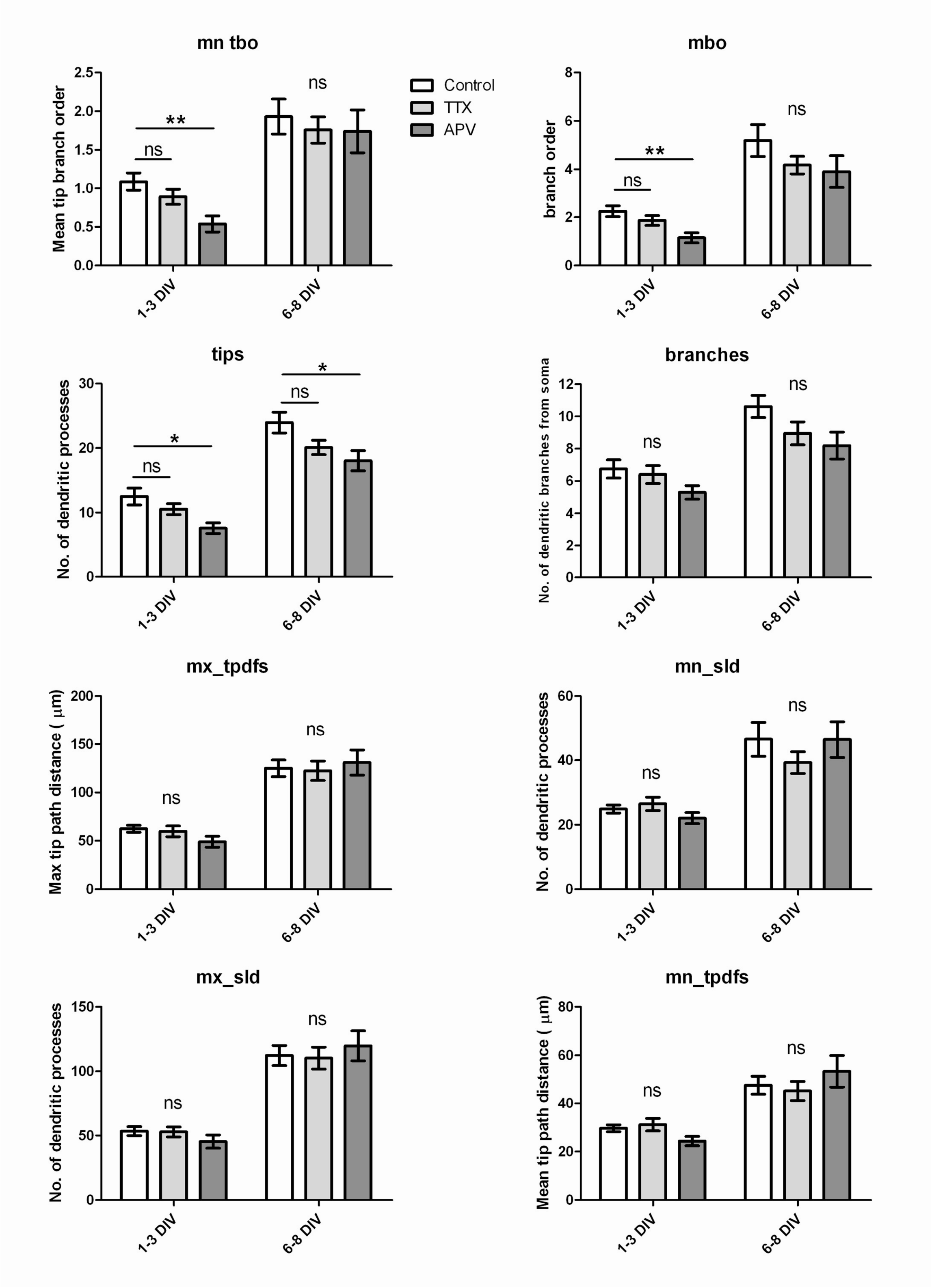

Figure S3, related to Figure 5. Quantification of other aspects of dendritic arbor formation following 48 hrs of TTX or APV treatment at different developmental timepoints. Error

bars represent sem. mn tbo: mean tip branch order, mbo: maximum branch order of any tip (eg. 0=soma branch, 1=1st bifurcation, etc.), tips: number of dendritic processes, branches: number of soma branches, mx_tpdfs: maximum distance from any tip back to soma, along dendrites, mn_sld: mean distance from tip to soma, straight line, mx_sld: maximum distance from tip to soma, straight line, mn_tpdfs: mean distance from tip back to soma, along dendrites.
